# Proline‐rich transmembrane protein 2 specifically binds to GluA1 but has no effect on AMPA receptor‐mediated synaptic transmission

**DOI:** 10.1002/jcla.24196

**Published:** 2022-01-08

**Authors:** Hao‐Yang Feng, Fengchang Qiao, Jianxin Tan, Xiaozuo Zhang, Ping Hu, Yun Stone Shi, Zhengfeng Xu

**Affiliations:** ^1^ Department of Prenatal Diagnosis Women's Hospital of Nanjing Medical University Nanjing Maternity and Child Health Care Hospital Nanjing China; ^2^ Ministry of Education Key Laboratory of Model Animals for Disease Study Model Animal Research Center Medical School Nanjing University Nanjing China; ^3^ State Key Laboratory of Pharmaceutical Biotechnology Department of Neurology Affiliated Drum Tower Hospital Medical School Nanjing University Nanjing China; ^4^ Institute for Brain Sciences Nanjing University Nanjing China; ^5^ Chemistry and Biomedicine Innovation Center Nanjing University Nanjing China

**Keywords:** AMPA receptor, GluA1, mental retardation, PRRT2, whole‐exome sequencing

## Abstract

**Background:**

Proline‐rich transmembrane protein 2 (PRRT2) is a neuron‐specific protein associated with seizures, dyskinesia, and intelligence deficit. Previous studies indicate that PRRT2 regulates neurotransmitter release from presynaptic membranes. However, PRRT2 can also bind AMPA‐type glutamate receptors (AMPARs), but its postsynaptic functions remain unclear.

**Methods and results:**

Whole‐exome sequencing used to diagnose a patient with mental retardation identified a nonsense mutation in the *PRRT2* gene (c.649C>T; p.R217X). To understand the pathology of the mutant, we cloned mouse *Prrt2* cDNA and inserted a premature stop mutation at Arg223, the corresponding site of Arg217 in human PRRT2. In mouse hippocampal tissues, Prrt2 interacted with GluA1/A2 AMPAR heteromers but not GluA2/A3s, via binding to GluA1. Additionally, Prrt2 suppressed GluA1 expression and localization on cell membranes of HEK 293T cells. However, when Prrt2 was overexpressed in individual hippocampal neurons using in utero electroporation, AMPAR‐mediated synaptic transmission was unaffected. Deletion of Prrt2 with the CRIPR/Cas9 technique did not affect AMPAR‐mediated synaptic transmission. Furthermore, deletion or overexpression of Prrt2 did not affect GluA1 expression and distribution in primary neuronal culture.

**Conclusions:**

The postsynaptic functions of Prrt2 demonstrate that Prrt2 specifically interacts with the AMPAR subunit GluA1 but does not regulate AMPAR‐mediated synaptic transmission. Therefore, our study experimentally excluded a postsynaptic regulatory mechanism of Prrt2. The pathology of PRRT2 variants in humans likely originates from defects in neurotransmitter release from the presynaptic membrane as suggested by recent studies.

## Graphical abstract

Prrt2 protein can specifically bind to GluA1 subunit, rather than GluA2 or GluA3. However, when Prrt2 was overexpressed in individual hippocampal neurons using in utero *electroporation*, AMPAR‐mediated synaptic transmission was unaffected. Deletion of Prrt2 with the CRIPR/Cas9 technique did not affect AMPAR‐mediated synaptic transmission

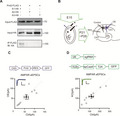

## 1 INTRODUCTION

Since its first pathogenic mutation was identified in humans in 2011, proline‐rich transmembrane protein 2 (*PRRT2*) (chromosome 16p11.2) has been considered the causative gene for several neurologic diseases, such as paroxysmal kinesigenic choreoathetosis, benign familial infantile epilepsy, and familial infantile convulsions with paroxysmal choreoathetosis.^1^, ^2^, ^3^ PRRT2, which is selectively expressed in neurons and located at synapses, plays a crucial role in neuronal migration, spinogenesis, and synapse formation and maintenance during development. Previously, researchers have revealed its interaction with SNAP25, a plasma membrane SNARE protein, and Syts1 and 2, Ca^2+^ sensors that mediate neurotransmitter release, suggesting that PRRT2 comprises a substantial component of the neurotransmitter release machinery at presynaptic terminals.^4^, ^5^

Although most studies have focused on PRRT2 function at the presynaptic membrane,^6^, ^7^ scattered evidence suggests that PRRT2 may regulate glutamate receptor function at the postsynaptic membrane. Prrt2 has been detected in postsynaptic densities in rodents, although at lower levels than found in presynaptic densities.^4^ In 2014, Schwenk et al. reported a list of dozens of proteins that bind native α‐amino‐3‐hydroxy‐5‐methyl‐4‐isoxazole‐propionicacid receptors (AMPARs), which included Prrt2.^8^ Subsequently, interaction of Prrt2 with AMPARs was verified in vitro and in vivo.^9^

Here, we report the case of a patient carrying a *PRRT2* mutant (c.649C>T; p.R217X) who had clinical manifestations of mental retardation. Transfection of Prrt2_R223X, a mimicking mutant from mouse Prrt2, in HEK 293T cells showed that the mutation led to the loss of Prrt2 protein. We analyzed the effects of Prrt2 on AMPARs with in vivo and in vitro systems and found that Prrt2 specifically bound to GluA1 but not the GluA2 or GluA3 subunits. In HEK 293T cells, Prrt2 suppressed the total protein level and localization of co‐transfected GluA1. However, in hippocampal CA1 neurons, neither overexpression nor deletion of Prrt2 affected GluA1 expression or synaptic AMPAR function. Thus, we conclude that Prrt2 does not regulate AMPAR function in vivo.

## 2 MATERIALS AND METHODS

### 2.1 Animals

All experiments were performed in accordance with established protocols (certificate number: AP#SY06) approved by the Institutional Animal Care and Use Committees of Nanjing University. Three litters of *C57BL*/*6JGpt* mice (age, 0–42 days) were used for age‐dependent Prrt2 expression analysis. Six female ICR mice (age, 4–6 weeks; weight, 50‐60g) were utilized for in utero electroporation (IUE). Five Cas9‐knock‐in (*B6*/*JGpt*‐*Rosa26^tm1(CAG^* ^−^ *^Cas9^* ^−^ *^tdTomato)^*/*Gpt*) mice and 6 *C57BL*/*6JGpt* mice (age, 0 days) were used for primary neuron culture. All mice were purchased from the Model Animal Research Center (Nanjing University). Mice were housed in pathogen‐free conditions at 22°C, 55% relative humidity, and under a 12‐h light/dark cycle, with provision of food and water ab libitum.

### 2.2 Experimental constructs

The cDNAs of mouse GluA1, GluA2, GluA3, and Prrt2 were used in this study. The HA‐tagged and FLAG‐tagged recombinant proteins used for Western blotting were generated by overlapping PCR (Vazyme Biotech, P505) and subcloned into the pCAGGS vector. An HA‐tag was attached to the N‐terminals of GluA1, GluA2, and GluA3 and was used for Western blot detection of these proteins. To generate the *Prrt2* c.667C>T (p.R223X) construct, we performed site‐directed mutagenesis by PCR using the wild‐type (WT) vector. Mutant constructs were confirmed by sequencing over the entire length of the coding region.

Design and screening of single‐guide (sg)RNAs for the clustered regularly interspaced short palindromic repeats (CRISPR) constructs were performed as previously described.^10^ The Prrt2 sgRNA was designed to target part of the coding region of exon 2. The primers used were as follows: 5′‐ACCGTTCAGCCGGGCCCAGGCATC‐3′ (forward) and 5′‐AAACGATGCCTGGGCCCGGCTGAA‐3′ (reverse). The sgRNA expression vector was constructed by inserting the in vitro synthesized PRRT2 sgRNA‐targeted sequence into a vector that contained a tracrRNA sequence, and expression of the fused sgRNA was driven by the U6 promoter. After verifying the efficiency of the system, spCas9 was subcloned into the preceding vector.

### 2.3 Cell culture

HEK 293T cells (ATCC) were cultured in Dulbecco's modification of Eagle's medium (Gibco, Thermo) containing 10% FBS (Gibco, Thermo) at 37°C and 5% CO_2_ and changed every 2 days. The primary hippocampal neurons were obtained from postnatal day 0 (P0) mice and cultured in Neurobasal (Gibco, Thermo) containing 2% B27 (Gibco, Thermo) and 1% Glutamax (Gibco, Thermo) at 37°C and 5% CO_2_ and changed every 3 days.

### 2.4 Co‐immunoprecipitation

HEK 293T cells were co‐transfected with the indicated expression plasmids in 10‐cm dishes 48 h before use. Cells were washed three times with phosphate‐buffered saline (PBS), harvested, and solubilized in co‐immunoprecipitation assay lysis buffer (Bio TeKe Corporation, Shanghai, China) and 1 mM phenylmethylsulfonyl fluoride for 1 h at 4°C. After centrifugation at 13,800 × *g* for 20 min, the pellet was discarded. Lysates were then incubated with antibodies at 4°C overnight. Then, the lysates were incubated with Protein G beads (GE Healthcare, USA) for 2 h at 4°C on a rotating platform. After incubation, the beads were washed four times with lysis buffer and boiled in 40 μl of 2× Laemmli buffer.

### 2.5 Western blots

HEK 293T cells were transiently transfected using the Lipofectamine 2000 reagent (Invitrogen) following the manufacturer's instructions. The basal DMEM medium without FBS was replaced 2 h before transfection, and 10% FBS was changed back to 6 h after transfection. Before cell harvest, the fluorescence would be detected to verify the success of transfection. Then, the cells were lysed in RIPA buffer containing 150 mM NaCl, 50 mM Tris (pH 7.4), 1% Nonidet P‐40, 0.5% sodium deoxycholate, and a mixture of protease inhibitors (Roche, Switzerland). After incubation at 4 °C for 60 min, the cell lysates were centrifuged for 30 min at 13,800 × *g* at 4°C. Then, the supernatant was mixed with 5× loading buffer and dithiothreitol and boiled. To detect full‐length receptors, the mixture was immediately loaded onto 10% sodium dodecyl sulfate‐polyacrylamide gel electrophoresis gels. The protein bands were transferred to polyvinylidene fluoride membranes (Millipore) at 100 V for 2 h and then blocked in 5% non‐fat milk dissolved in tris‐buffered saline with Tween 20 at room temperature for 1 h. Finally, the GluA1, GluA2, and GluA3 receptor subunits were probed with anti‐GluA1 (Abcam, polyclonal, rabbit anti‐mouse, Cat. No. ab31232, 1:10,000), anti‐GluA2 (Abcam, monoclonal, rabbit anti‐mouse, Cat. No. ab133477, 1:10,000), and anti‐GluA3 (CST, monoclonal, rabbit anti‐mouse, Cat. No. 4676, 1:2000) antibodies, respectively, and Prrt2 was probed with an anti‐Prrt2 antibody (Sigma, polyclonal, rabbit anti‐mouse, Cat. No. HPA014447, 1:2000). Proteins were detected by addition of an enhanced chemiluminescence substrate (Thermo) before exposure. Anti‐HA (CST, rabbit monoclonal, Cat. No. 3724, 1:1000) and anti‐FLAG (CST, rabbit polyclonal, Cat. No. 2368T, 1:1000) antibodies were employed for co‐immunoprecipitation assays. The internal reference protein antibodies used included anti‐GAPDH (Bioworld, monoclonal, mouse anti‐mouse, Cat. No. MB001, 1:10,000), anti‐IGF1R (Proteintech, polyclonal, rabbit anti‐mouse, Cat. No. 20254–1‐AP, 1:1000), anti‐β‐Tubulin (Sigma, mouse monoclonal, Cat. No. T8660, 1:2000), and anti‐mCherry (Abcam, rabbit polyclonal, Cat. No. ab167453, 1:1000).

### 2.6 Extraction of membrane proteins

HEK 293T cells were cultured and transfected with the indicated constructs. After 48 h, the cultured cells were washed twice with ice‐cold PBS and incubated in PBS containing 1 mg/ml Sulfo‐NHS‐SS‐Biotin (Thermo) for 0.5–1 h at 4°C with mild shaking. The biotinylation reaction was quenched, and unbound biotin was removed by washing the cells twice with PBS‐Ca‐Mg containing 100 mM glycine for 15 min at 4°C. The cells were then lysed with lysis buffer. The supernatants were collected and incubated with streptavidin beads (Thermo) overnight at 4°C, then washed four times with lysis buffer, and eluted using 2× Laemmli sample buffer.

### 2.7 In utero electroporation

Embryonic day 15 (E15) pregnant mice were anesthetized with 1% pentobarbital sodium (dissolved in normal saline) with 100 μl per 10 g mice dose by peritoneal injection before surgery.^11^, ^12^ To visualize the electroporating process, plasmids were mixed with 2 mg/ml Fast Green (Sigma‐Aldrich), and pCAG‐U6‐sgRNA‐hUbc‐spCAC9‐T2A‐GFP was used at a final concentration of 2 μg/μl. During surgery, the uterine horns were exposed, and one lateral ventricle of each embryo was pressure injected with 1–2 μl of plasmid DNA. Injections were performed by inserting a pulled glass microelectrode into the lateral ventricle through the uterine wall and embryonic membranes and injecting the content of the microelectrode by pressure. The embryos were then electroporated with five 50 ms, 40 V pulses delivered at 1 Hz using platinum Tweezertrodes with a square‐wave pulse generator (BTX, Harvard Apparatus). Following electroporation, the embryos were placed back into the abdominal cavity, and the muscle and skin were sutured. The pregnant mice were then allowed to recover from surgery, and the pups were normally delivered. The full gestation period of each pregnant mouse is 19–21 days. All maternal mice that suffered with IUE were recovered from the surgery, and pups were delivered naturally. The maternal mice were humanely euthanized with CO_2_ at the end of the nursing period. Death was confirmed by observing respiration and by using the corneal reflection method.

### 2.8 Electrophysiology

Voltage‐clamp recordings were performed on CA1 pyramidal neurons in acute hippocampal slices. The acute hippocampal slices were obtained from mice anesthetized (1% pentobarbital sodium) and decapitated at 21–28 days after IUE. To prepare acute slices, 300‐μm transverse slices were cut using a Leica vibratome (Leica VT1000S) in chilled high‐sucrose cutting solution containing the following (in mM): 2.5 KCl, 0.5 CaCl_2_, 7 MgCl_2_, 1.25 NaH_2_PO_4_, 25 NaHCO_3_, 7 D‐glucose, 210 sucrose, and 1.3 ascorbic acid. The slices were then incubated for 30 min at 34°C in artificial cerebrospinal fluid (ACSF) containing the following (in mM): 119 NaCl, 2.5 KCl, 26.2 NaHCO_3_, 1 NaH_2_PO_4_, 2.5 CaCl_2_, 1.3 MgSO_4_, and 11 D‐glucose and bubbled with 95% O_2_ and 5% CO_2_ to maintain pH. The slices were allowed to recover at room temperature for 30 min to 1 h before recording. For recording of excitatory transmission, slices were transferred to a perfusion stage and perfused with ACSF containing 0.1 mM picrotoxin and 0.01 mM bicuculline. Synaptic responses were evoked by stimulating the stratum radiatum of the CA1 region with a bipolar metal electrode. To ensure stable recording, the membrane holding current, input resistance, and pipette series resistance were monitored throughout the recording. Data were collected using a MultiClamp 700B amplifier (Axon Instruments, Molecular Devices), filtered at 2 kHz, and digitized at 10 kHz.

### 2.9 Whole‐cell synaptic recordings

Simultaneous dual whole‐cell recordings were performed on green fluorescent protein (GFP)‐positive experimental cells as identified by epifluorescence and neighboring nontransfected control cells. The internal recording solution contained the following (in mM): 135 CsMeSO_4_, 8 NaCl, 10 HEPES, 0.3 EGTA, 5 QX314‐Cl, 4 Mg‐ATP, 0.3 Na‐GTP, and 0.1 spermine. The osmolarity was adjusted to 290–295 mOsm, and the pH was buffered at 7.3–7.4. AMPAR‐mediated responses were isolated by voltage‐clamping the cell at −70 mV, whereas N‐methyl‐D‐aspartic acid receptor (NMDAR)‐mediated responses were recorded at +40 mV, with amplitudes measured 100 ms after stimulation to avoid contamination by AMPAR current.

### 2.10 Immunofluorescence

Cultured primary mouse neurons were transfected with Lipofectamine 2000 at 2 days in vitro (DIV2) and harvested at DIV20. Then, the neurons were fixed in 4% paraformaldehyde, blocked and permeabilized in 5% bovine serum albumin (BSA) in PBS containing 0.3% Triton‐X, and stained with primary antibodies against Prrt2 (Sigma, Cat. No. HPA014447, 1:500) and GFP (Abcam, chicken polyclonal, Cat. No. ab13970, 1:500), followed by washing with PBST and staining with Alexa 488/549‐conjugated secondary antibodies. For GluA1 intracellular and surface immunofluorescence, the neurons were first blocked in 5% BSA and then stained with an antibody against GluA1 (Abcam, Cat. No. ab31232, 1:500) and an Alexa 649‐conjugated secondary antibody. Next, the neurons were permeabilized with 0.3% Triton‐X and stained with antibodies against GluA1‐NT (Millipore, mouse monoclonal, Cat. No. MAB2263, 1:500) and GFP, followed by staining with Alexa 488/549‐conjugated secondary antibodies.

### 2.11 Statistical analysis

Normalization was performed by dividing both the control and experimental conditions by the average value of the control. The paired whole‐cell data were analyzed using the two‐tailed Wilcoxon signed‐rank test, and unpaired data using the Mann‐Whitney U test. The one‐way ANOVA test for multiple comparisons was used to analyze all the other experiments involving unpaired data. Data analysis was performed using Excel (Microsoft) and GraphPad Prism (GraphPad Software).

### 2.12 Consent

Written informed consent to participate in this study was obtained from the patient and his parents. All procedures were approved by the Institutional Review Board (Ethics Committee) of Nanjing Maternity and Child Health Care Hospital ([2017] KY‐081).

## 3 RESULTS

### 3.1 Case report of the *PRRT2* c.649C>T mutant

**FIGURE 1:**
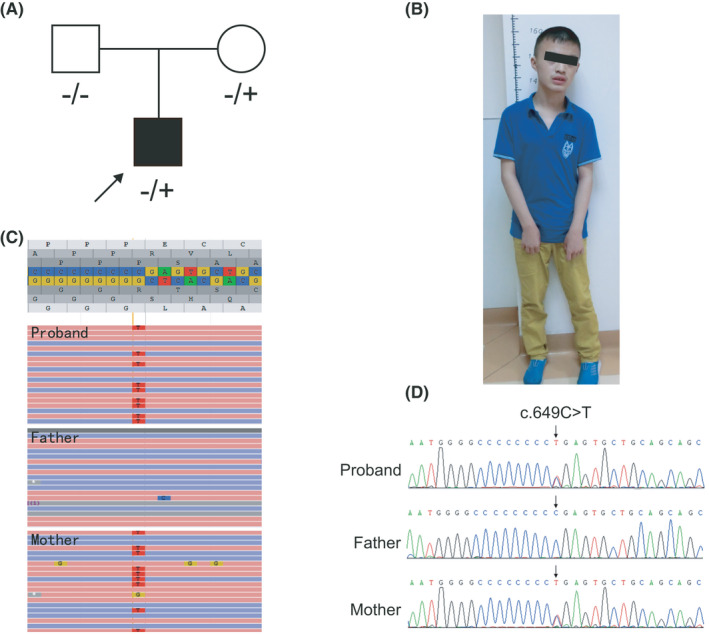
Pedigree and mutation analysis of the family. (A) Pedigree of the family. (B) Clinical features of our patient show clinical manifestation of mental retardation. (C) WES results showed that the proband carried a heterozygous nonsense mutation (c.649C>T), which was also found in his mother. (D) Sanger sequencing results correspond to the WES results

The proband, a 14‐year‐old male patient from Jiangsu Province, was admitted to our center for genetic counseling and further evaluation in July 2017 with paroxysmal epilepsy and dysgnosia. The patient was delivered vaginally, and his first epilepsy event occurred at 5 months after birth, with normal brain computed tomography and electroencephalography results (Yancheng Hospital). At the age of 3 years, he was diagnosed with autism in Jiangsu Province Hospital. Afterward, the patient had occasional seizures, generally triggered by standing up suddenly and lasting only a few seconds, accompanied by dyskinesia. During the admission, the patient exhibited a dull countenance, an uneven standing position, unnatural curling of the hands, slow responses, a minor communication disorder, speech impairment, and poor emotional management (Figure 1A, B). According to his parents, he could not take care of himself well and lacked creative imagination. His IQ was 70.

Using whole‐exome sequencing, a heterozygous nonsense variant (Chr16:29825024 C>T; c.649C>T; p.R217X, NM_145239.3) in the coding region of exon 2 of the *PRRT2* gene was identified in the proband. Then, these results were verified by Sanger sequencing (Figure 1C, D). The same mutation was also found in his mother. Nevertheless, his mother, also a carrier of the heterozygous mutation, did not show similar symptoms.

### 3.2 The mutation causes loss of protein expression

**FIGURE 2:**
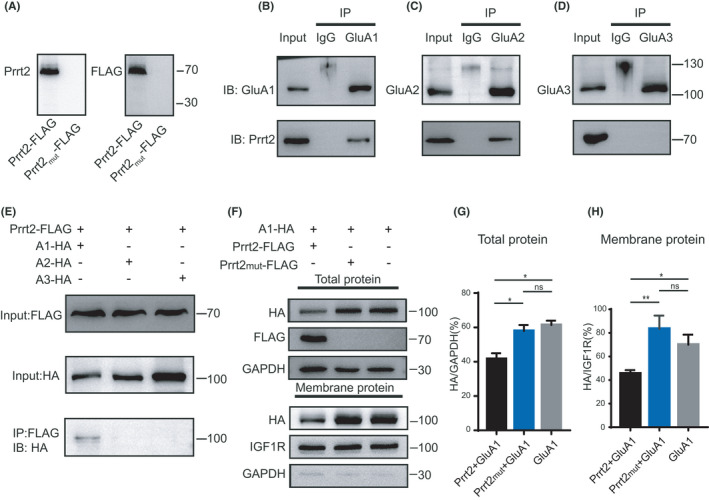
Interaction of Prrt2 with the GluA1 subunit in vivo and in vitro. (A) Protein levels of the truncated Prrt2‐FLAG plasmid (p.R223X, the corresponding mutant of p.R217X in human PRRT2) in HEK 293T cells. Antibodies against the N‐terminus of Prrt2 and FLAG were used to detect Prrt2‐FLAG. Western blotting results demonstrated that the mutant Prrt2 protein is undetectable. (B, C, D) In vivo co‐immunoprecipitation assays using the adult mouse hippocampus. After pull‐down with GluA1, GluA2, and GluA3 antibodies, Western blotting results demonstrated interactions between Prrt2 and both GluA1 and GluA2, but not GluA3. (E) In vitro co‐immunoprecipitation using cell extracts from HEK 293T cells co‐transfected with HA‐tagged GluA1, GluA2, and GluA3 and FLAG‐tagged Prrt2. After pull‐down with the FLAG antibody, Western blotting results demonstrated direct interactions between Prrt2 and GluA1. (F) Total and membrane proteins were extracted from HEK 293T cells co‐transfected with HA‐tagged GluA1, FLAG‐tagged Prrt2, and FLAG‐tagged mutant Prrt2 (p.R223X). (G) Western blotting results demonstrated decreased total amounts of GluA1 after co‐transfection with Prrt2 (*p = 0.024 Prrt2 + GluA1 vs. Prrt2_mut_ + GluA1; *p = 0.010 Prrt2 + GluA1 vs. GluA1; ns. *p *= 0.729 Prrt2_mut_ + GluA1 vs. GluA1; *n* = 3). (H) Western blotting results demonstrated decreased surface expression levels of GluA1 after co‐transfection with Prrt2 (**p = 0.003 Prrt2 + GluA1 vs. Prrt2_mut_ + GluA1; *p = 0.025 Prrt2 + GluA1 vs. GluA1; ns. *p *= 0.195 Prrt2_mut_ + GluA1 vs. GluA1; *n* = 3)

PRRT2 is highly conserved in mammals. To understand the pathology of the mutation found in our patient, we cloned mouse Prrt2 and introduced an R223X mutation to mimic that found in human PRRT2. A FLAG epitope was added to the C‐termini of WT and mutant Prrt2 to facilitate protein detection. When transfected in HEK 293T cells, both the Prrt2 and FLAG signals were undetectable in the cells that expressed mutant Prrt2, in sharp contrast to cells that expressed WT Prrt2. These results suggest that the truncation mutation leads to loss of Prrt2 expression, consistent with a previous study reporting that truncated Prrt2 is unstable or not expressed^13^ (Figure 2A).

### 3.3 Prrt2 specifically interacts with GluA1

To investigate the endogenous expression pattern of Prrt2 in various developmental stages, hippocampus tissues from mice on P0 to P42 were homogenized and incubated with a Prrt2 antibody. β‐Tubulin, a housekeeping gene, was used as an internal control. Western blotting analysis indicated that Prrt2 expression gradually increased from a low level at birth and reached a plateau at P14 (Figure S1A, B). Next, we examined the interaction between Prrt2 and AMPARs. Co‐immunoprecipitation experiments were performed with homogenates from the adult mouse hippocampus. We examined the ability of Prrt2 to bind to GluA1, GluA2, and GluA3 because they comprise the majority of AMPAR subunits in the hippocampus.^14^, ^15^ We found that both the GluA1 antibody and GluA2 antibody pulled down Prrt2 (Figure 2B, C). Conversely, GluA3 did not interact with Prrt2 (Figure 2D). Because AMPARs in the hippocampus mainly comprise heteromeric tetramers of GluA1/A2 or GluA2/A3,^15^ these data indicated that GluA1/A2, but not GluA2/A3, interacts with Prrt2. If this prediction is correct, then pull‐down of Prrt2 by GluA2 could occur via mediation of GluA1.

We then studied the interaction of Prrt2 with AMPAR subunits in HEK 293T cells. FLAG‐tagged Prrt2 was co‐expressed with GluA1, GluA2, and GluA3 tagged with an HA epitope at the N‐terminus following the signal peptides. As predicted, the co‐immunoprecipitation results showed that GluA1 interacted with Prrt2, while GluA2 or GluA3 did not interact (Figure 2E). These results verified our prediction that, in hippocampal tissue, GluA2 would indirectly pull down Prrt2 via GluA1.

### 3.4 Prrt2 suppresses GluA1 protein expression levels in vitro

We next examined the effects of Prrt2 on AMPAR expression. HA‐tagged GluA1 was co‐transfected with WT and mutant Prrt2 into HEK 293T cells. After 3 days of expression, biotin was used to label surface proteins. HA signals from whole‐cell homogenates and biotin‐labeled membrane proteins were analyzed to determine the total and surface GluA1 content, respectively. Compared with the control group expressing HA‐tagged GluA1 alone, Prrt2 suppressed total protein levels of GluA1, while co‐transfection of Prrt2_R223X did not suppress GluA1 (Figure 2F, G). Meanwhile, the surface expression level of GluA1 was also decreased after co‐transfection with Prrt2, consistent with a previous report^9^ (Figure 2F, H). In contrast, co‐expression of Prrt2 had no effect on the total and surface expression levels of GluA2 (Figure S1C–E), consistent with the observation that Prrt2 specifically interacts with GluA1. These results demonstrated that Prrt2 suppresses GluA1 expression in vitro.

### 3.5 Overexpression of Prrt2 does not affect synaptic AMPAR function

**FIGURE 3:**
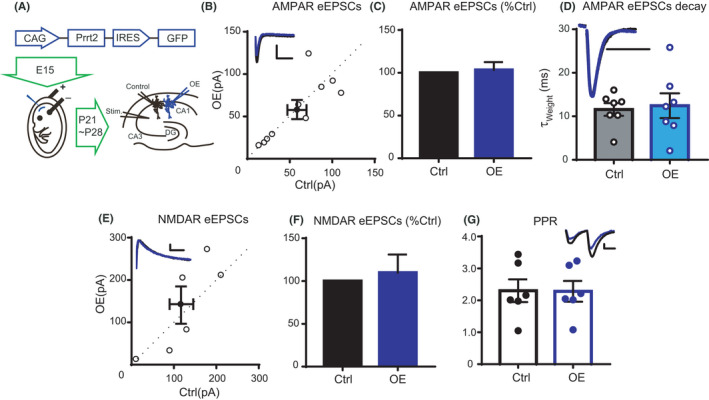
Overexpression of Prrt2 in the mouse hippocampal CA1 region. (A) Map of the Prrt2 plasmid used in the experiment (top), a schematic diagram of the experimental in utero electroporation procedures used in embryonic day 15 mice (left), and a schematic of the dual cell recording experiment approach (right). (B) The scatterplot shows amplitudes of AMPA receptor (AMPAR)‐evoked excitatory postsynaptic currents (eEPSCs) for single pairs (open circles) of control and transfected cells overexpressing Prrt2 (OE) (*n* = 10 pairs). The filled circle indicates the mean±SEM (B, Control = 58.4 ± 11.2; OE = 58 ± 11.6, pA). (C) Bar graph of ratios normalized to the control (%) summarizing the mean ± *SEM* of AMPAR eEPSC values represented in B (103.3 ± 9.2, *p *= 0.72). (D) Histogram showing statistical comparisons of the AMPAR eEPSC decay (*n* = 7 pairs). Hollow circles indicate τ of single samples (mean ± SEM, Control = 11.57 ± 1.47; OE = 12.43 ± 2.85, ms, *p *= 0.79). (E) The scatterplot shows amplitudes of NMDA receptor (NMDAR) eEPSCs for single pairs (open circles) of control and transfected cells overexpressing Prrt2 (OE) (*n* = 6 pairs). The filled circle indicates the mean±SEM (E, Control = 122.9 ± 28.6; OE = 137 ± 43.9, pA). (F) Bar graph of ratios normalized to the control (%) summarizing the mean ± SEM of NMDAR eEPSC values represented in E (109.8 ± 21.2, *p *= 0.65). (G) Histogram showing statistical comparisons of the paired‐pulse ratio (PPR) (*n* = 6 pairs). Filled circles indicate the ratios of single samples (mean ± SEM, Control = 2.30 ± 0.36; OE = 2.28 ± 0.32, *p *= 0.97). Scale bars: 100 ms, 50 pA

After characterization of the interaction of Prrt2 with AMPARs in vitro, we then studied its effects on synaptic AMPAR function. We first overexpressed Prrt2 in individual hippocampal neurons through IUE. In brief, female ICR mice underwent surgical operations to expose the uterus at 15 days of pregnancy. Prrt2‐internal ribosomal entry site‐GFP vectors were injected into the lateral ventricle of the pups and transfected into the hippocampus via electroporation (Figure 3A). The pregnant mice were then allowed to recover, and the pups were delivered. Hippocampal slices were prepared from pups at the age of 3–4 weeks. CA1 pyramidal neurons were sparsely labeled with GFP, indicating expression of transfected Prrt2. Typically, the cell responses of a Prrt2 overexpressing neuron (GFP‐positive) and an adjacent control neuron (GFP‐negative) were simultaneously recorded by stimulating a common pathway of the Schaffer collateral. AMPAR‐excitatory postsynaptic currents (EPSCs) were recorded by holding neurons at −70 mV. The postsynaptic currents were also recorded at +40 mV, at which potential the currents were mediated by both AMPARs and NMDARs. The NMDAR‐EPSCs were measured at 100 ms after stimulation, when the AMPAR‐EPSCs were decreased because of fast deactivation. In these experiments, we found that the amplitude and decay kinetics of evoked AMPAR‐EPSCs were not different between Prrt2 overexpressing and control neurons (Figure 3B–D). Meanwhile, NMDAR‐EPSCs were also unaltered by Prrt2 overexpression (Figure 3E, F). The paired‐pulse ratio (PPR), a measure of neurotransmitter release, was also unaltered, which suggests that overexpression of Prrt2 in postsynaptic neurons does not affect presynaptic glutamate release (Figure 3G). Because none of the examined excitatory transmissions were altered by IUE transfection of Prrt2, we speculated that the Prrt2 expression level had been altered in the transfected neurons. To test this possibility, we transfected the Prrt2 vector into primary culture of hippocampal neurons and found that immunofluorescence labeling of Prrt2 was significantly increased in Prrt2 transfected neurons (Figure S2A–C), demonstrating that transfected Prrt2 was stably expressed in these neurons.

### 3.6 Deletion of Prrt2 does not affect synaptic AMPAR function in neurons

**FIGURE 4:**
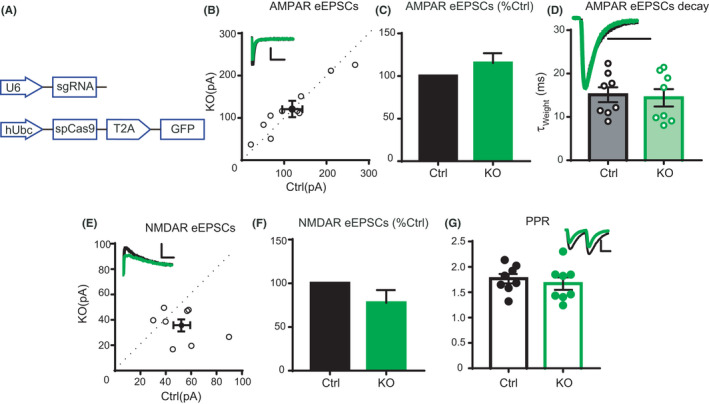
Deletion of Prrt2 in the mouse hippocampus CA1 region. (A) Map of the CRISPR construct used in the experiment. (B) The scatterplot shows amplitudes of AMPA receptor (AMPAR)‐evoked excitatory postsynaptic currents (eEPSCs) for single pairs (open circles) of control and transfected Prrt2‐knockout (KO) cells (*n* = 10 pairs). The filled circle indicates the mean ± *SEM* (B, Control = 118.8 ± 23.9; KO = 120.9 ± 19.4, pA). (C) Bar graph of ratios normalized to the control (%) summarizing the mean ± *SEM* of AMPAR eEPSC values represented in B (115.3 ± 11.6, *p *= 0.20). (D) Histogram showing statistical comparisons of the AMPAR eEPSC decay (*n* = 8 pairs). Hollow circles indicate the τ of single samples (mean ± SEM, Control = 15.12 ± 1.72; KO = 14.41 ± 1.99, ms, *p *= 0.79). (E) The scatterplot shows the amplitudes of NMDA receptor (NMDAR) eEPSCs for single pairs (open circles) of control and transfected KO cells (*n*=8 pairs). The filled circle indicates the mean ± *SEM* (E, Control = 52.4 ± 6.5; KO = 35.7 ± 4.6, pA); (F) Bar graph of ratios normalized to the control (%) summarizing the mean ± *SEM* of NMDAR eEPSC values represented in E (77.7 ± 14.6, *p *= 0.15). (G) Histogram showing statistical comparisons of the paired‐pulse ratio (PPR) (*n* = 8 pairs). Filled circles indicate the ratios of single samples (mean ± *SEM*, Control = 1.77 ± 0.09; KO = 1.67 ± 0.12, *p *= 0.54). Scale bars: 100 ms, 50 pA

There are two possible explanations for the lack of changes in synaptic function of AMPARs. One is that Prrt2 does not regulate AMPAR expression in neurons. Alternatively, the endogenous Prrt2 in hippocampal CA1 neurons could be saturated, and expression of additional Prrt2 does not exert an effect. To address these possibilities, we knocked out endogenous Prrt2 in hippocampal CA1 neurons using the CRISPR/Cas9 technique, which has been shown to efficiently delete targeting molecules in neurons.^16^ We developed a *Prrt2*‐knockout construct, CRISPR_*Prrt2*, containing both a *Prrt2*‐targeting sgRNA and Cas9. The Cas9 cDNA was fused with GFP by T2A sequence so that GFP signal represents Cas9 expression (Figure 4A).^17^ To verify the efficiency of CRISPR/Cas9 in knocking out the *Prrt2* gene, plasmids expressing sgRNA_targeting *Prrt2*, Cas9, and Prrt2‐FLAG were co‐transfected into HEK 293T cells. The control group was transfected with the Prrt2 plasmid alone. A negative sgRNA that does not target *Prrt2* was used as a negative control. Western blotting assays revealed that, compared with the control group, the level of Prrt2 in the test group was reduced by 70%, and the negative sgRNA exhibited no effect (Figure S2D, E). In cultured neurons isolated from hippocampi of the Cas9‐knock‐in mice, lentivirus‐mediated expression of *Prrt2* sgRNA nearly completely depleted Prrt2 (Figure S2F, G). These results demonstrated that the sgRNA was highly effective in eliminating Prrt2. Then, the constructed CRISPR_*Prrt2* plasmid was injected into ventricles of E15 mice, and hippocampal CA1 pyramidal neurons were transfected by IUE, as previously described (Figure 3A).

Simultaneous dual whole‐cell recordings from a transfected GFP‐positive cell and a neighboring control neuron showed that deletion of Prrt2 had no obvious effect on the amplitude of AMPAR‐EPSCs (Figure 4B, C). Furthermore, the decay kinetics of AMPAR‐EPSCs was also unaltered by Prrt2 deletion, indicating that the composition of synaptic AMPARs is not changed (Figure 4D). Meanwhile, neither NMDAR‐EPSCs nor the PPRs were altered by deletion of Prrt2 (Figure 4E–G). These results demonstrate that Prrt2 deletion in mouse CA1 neurons does not affect synaptic trafficking of AMPARs.

### 3.7 Overexpression or deletion of Prrt2 does not affect the surface/intracellular ratio of GluA1

**FIGURE 5:**
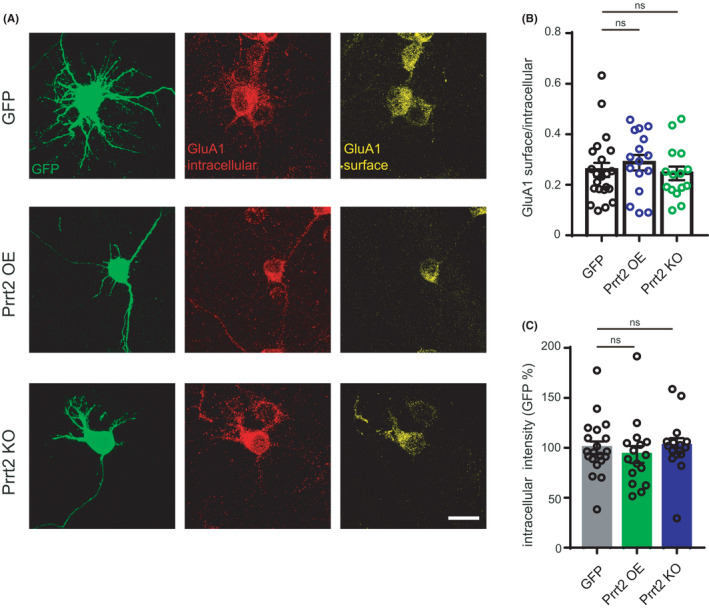
Immunofluorescence intensities of surface and intracellular GluA1. (A) Immunofluorescence staining of green fluorescent protein (GFP, green) and GluA1 (intracellular, red; surface, yellow) in primary hippocampal neurons transfected with GFP, overexpression (OE), or knockout (KO) plasmids; Scale bar: 20 μm. (B) Immunofluorescence intensity analysis of intracellular GluA1 (ns. *p *= 0.750 GFP vs. Prrt2 OE; ns. *p *= 0.944 GFP vs. Prrt2 KO). (C) Immunofluorescence intensity analysis of the surface/intracellular ratio (ns. *p *= 0.784 GFP vs. Prrt2 OE; ns. *p *= 0.976 GFP vs. Prrt2 KO)

Our electrophysiological analysis indicated that synaptic AMPARs are not altered by overexpression or deletion of Prrt2. Neurons may have an ability to stabilize synaptic receptors even when the overall expression level of AMPARs is altered. To test this possibility, we measured total GluA1 expression levels and the surface/intracellular ratio in primary culture of hippocampal neurons. Surface GluA1 was labeled with a mouse antibody against the extracellular domains of GluA1 in cultured neurons under conditions where cells were impermeable. Then, neurons were permeabilized with 0.3% Triton‐X, and intracellular GluA1 was labeled with a rabbit antibody. Immunofluorescence intensity analysis demonstrated that the surface/intracellular ratio of GluA1 was not different among GFP vector transfected, Prrt2 overexpressed and knockout neurons, suggesting that Prrt2 does not affect GluA1 trafficking in hippocampal neurons (Figure 5A, B). Furthermore, the fluorescence intensity of GluA1 labeling under the permeabilized condition was not different among the three groups (Figure 5C), suggesting that GluA1 expression is unaltered by manipulation of Prrt2.

## 4 DISCUSSION

In the present study, we characterized the effects of Prrt2 on the function of synaptic AMPARs. Biochemical and electrophysiological analyses revealed two main findings. First, Prrt2 specifically binds to the AMPAR subunit GluA1 but not to GluA2 or GluA3. Second, although Prrt2 suppresses GluA1 expression in HEK 293T cells, Prrt2 has no apparent effects on synaptic AMPARs.

In clinical practice, we identified a heterozygous nonsense mutant in the *PRRT2* gene (c.649C>T; p.R217X) in a male patient exhibiting classic epilepsy and paroxysmal dyspraxia phenotypes, similar to patients carrying *PRRT2* mutants reported previously.^1^, ^2^, ^3^ In addition to motor system disorders, this patient presented obvious clinical features of mental retardation, such as unnatural posture and poor learning ability and lack of creativity. Notably, the same mutant was reported once previously^18^ with similar motor disorders, while mental retardation was not mentioned. According to the literature, 52.4% of patients carrying homozygous *PRRT2* truncation mutations have intellectual disabilities, whereas only 0.6% of patients with heterozygous mutations have these symptoms,^2^ suggesting a gene‐dosage‐dependence of PRRT2 in intellectual development. Our patient may suffer from severe dosage deficiency even though he is a heterozygous mutation carrier. Intriguingly, his mother, who is also a heterozygous carrier of the same mutant, has never manifested any related symptoms. We suspect that his mother has incomplete penetrance, as cases of incomplete penetrance of *PRRT2* mutants have been previously reported.^1^

We cloned mouse Prrt2 and generated an R223X mutation to mimic PRRT2_R217X in humans. When Prrt2_R223X was expressed in HEK 293T cells, the Prrt2 protein level was undetectable, which is consistent with previous reports indicating that truncation mutants near R217 in PRRT2 were either unstable or not expressed.^13^ Yet, there is no experimental evidence to distinguish these two possibilities. Therefore, patients with the PRRT2_R217X mutation might have abnormal PRRT2 levels and may suffer from dosage deficiency.

Several lines of evidence indicate that Prrt2 can bind to AMPARs and may regulate AMPAR expression or function. In proteomic studies of the synaptic AMPAR complex, antibodies against GluA2 could pull down Prrt2 in mouse brain tissue.^8^, ^19^ A more recent study found that Prrt2 binds to GluA1 and suppresses its surface level when these two proteins are heterologously co‐expressed in HEK 293T cells.^9^ We found that antibodies against Prrt2 can pull down GluA1 and GluA2 but not GluA3 in mouse brain tissues. In HEK 293T cells, Prrt2 only interacted with GluA1 and not with GluA2 or GluA3. Therefore, we concluded that GluA2 pulled down Prrt2 indirectly through GluA1 in brain tissues. This finding is consistent with the notion that AMPARs in the cortex/hippocampus mainly exist in the GluA1/A2 and GluA2/A3 forms.^15^ Recently, observation of native AMPARs using cryo‐electron microscopy technology identified GluA1/A2/A3 type AMPARs in the brain.^20^ However, antibodies against Prrt2 failed to pull down GluA3, indicating that either Prrt2 does not bind to GluA1/A2/A3 type AMPARs or that the amount of this type of AMPAR is minimal in the brain.

A previous study has verified that WT PRRT2, but not its truncated mutants, suppresses the surface distribution of GluA1 in vitro.^9^ In the current study, we found that Prrt2 suppresses both total and surface GluA1 in HEK 293T cells, largely reconstituting previous observations.^9^ However, overexpression or deletion of Prrt2 in hippocampal neuronal culture had no obvious effects on GluA1 expression or membrane distribution. Manipulation of Prrt2 in CA1 pyramidal neurons in vivo also did not affect AMPAR‐EPSCs. AMPARs in hippocampal CA1 neurons are mostly GluA1/A2 heteromers, which is a slow type of AMPARs.^15^ If this GluA1/A2 is replaced by faster AMPARs such as GluA2/A3 (another component of AMPARs in CA1 neurons), then the decay kinetics of AMPAR EPSC will be speeded. We thus calculated the decay kinetics of AMPAR‐EPSCs and found no change (Figure 3D, Figure 4D), suggesting unaltered composition of synaptic AMPARs. Thus, Prrt2 appears to have no regulatory effects on postsynaptic AMPARs in vivo.

There is a possibility that even though Prrt2 does not regulate AMPAR function in rest condition, it may change activity‐dependent neuronal plasticity. We believe this is unlikely as any factor that has a role in neuronal plasticity, it generally affects basic transmission.^11^, ^21^, ^22^, ^23^

There are several possible explanations as to why Prrt2 suppresses GluA1 in HEK 293T cells but not in AMPARs in neurons. First, the expression of GluA1 in HEK 293T cells cannot fully mimic GluA1/A2 in neurons. Second, many other AMPAR binding proteins exist such as transmembrane AMPAR regulatory proteins and cornichons.^21^, ^24^ These factors may impose stronger regulation on AMPARs, which may overwhelm the effects of Prrt2. Third, neurons might have stronger regulatory capability than HEK 293T cells. For instance, it is assumed that Prrt2 facilitates GluA1 degradation in HEK 293T cells, while degradation of AMPARs is strongly controlled by factors other than Prrt2. Therefore, overexpression or deletion of Prrt2 produces little effect.

Overall, our study demonstrates that although it specifically binds to the GluA1 subunit, Prrt2 is not involved in regulating surface trafficking or basic transmission of AMPARs in hippocampal CA1 neurons. It would be of interest to learn whether Prrt2 exhibits postsynaptic regulation of AMPARs in interneurons or neurons in other brain regions. It should be noted that our conclusion was based on overexpression and deletion of mouse neurons. Whether this conclusion can be fully extend to human or the patient needs to be verified in the future.

## CONFLICT OF INTEREST

The authors declare no conflict of interest.

## AUTHOR CONTRIBUTIONS

H.Y.F. carried out the experiments. F.C. contributed to Figure 1. P.H., Y.S.S., and Z.X. supervised the study. Y.S.S., J. T., and H.Y.F. wrote the article.

## Supporting information

Fig S1‐S2
