# Supplementary material for: Proline‐rich transmembrane protein 2 specifically binds to GluA1 but has no effect on AMPA receptor‐mediated synaptic transmission
Source: J Clin Lab Anal. 2022 Jan 8;36(2):e24196. doi: 10.1002/jcla.24196 (PMC8842155; doi:10.1002/jcla.24196)
Supplement: Supplementary file 1 — Fig S1‐S2 [file JCLA-36-e24196-s001.docx]

**Supplementary Materials:**


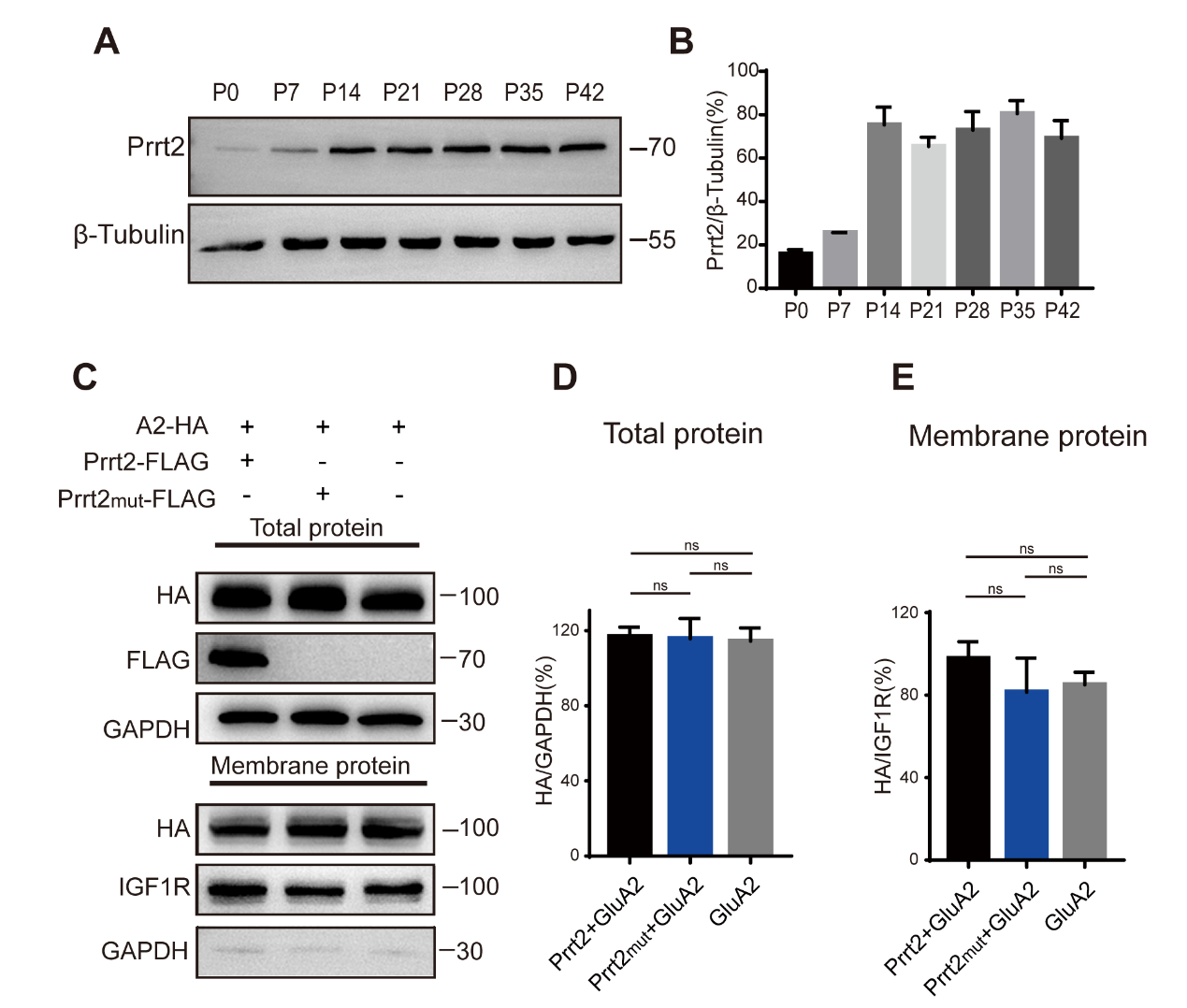


**Figure S1.** The expression levels of Prrt2. **(A)** Protein level of Prrt2 in the postnatal day 0 (P0) to P42 mouse hippocampus. **(B)** Western blotting results demonstrated that the Prrt2 level increased and reached a plateau at P14 in the mouse hippocampus. **(C)** Total and membrane proteins were extracted from HEK 293T cells co-transfected with HA-tagged GluA2, FLAG-tagged Prrt2, and FLAG-tagged mutant Prrt2 (p.R223X). **(D)** Western blotting results demonstrated unaltered total amounts of GluA2 after co-transfection with Prrt2 (ns. P=0.994 Prrt2+GluA2 vs. Prrt2_mut_+GluA2; ns. P=0.973 Prrt2+GluA2 vs. GluA2; ns. P=0.993 Prrt2_mut_+GluA2 vs. GluA2; n=3). **(E)** Western blotting results demonstrated unaltered membrane surface levels of GluA2 after co-transfection with Prrt2 (ns. P=0.589 Prrt2+GluA2 vs. Prrt2_mut_+GluA2; ns. P=0.716 Prrt2+GluA2 vs. GluA2; ns. P=0.973 Prrt2_mut_+GluA2 vs. GluA2; n=3).


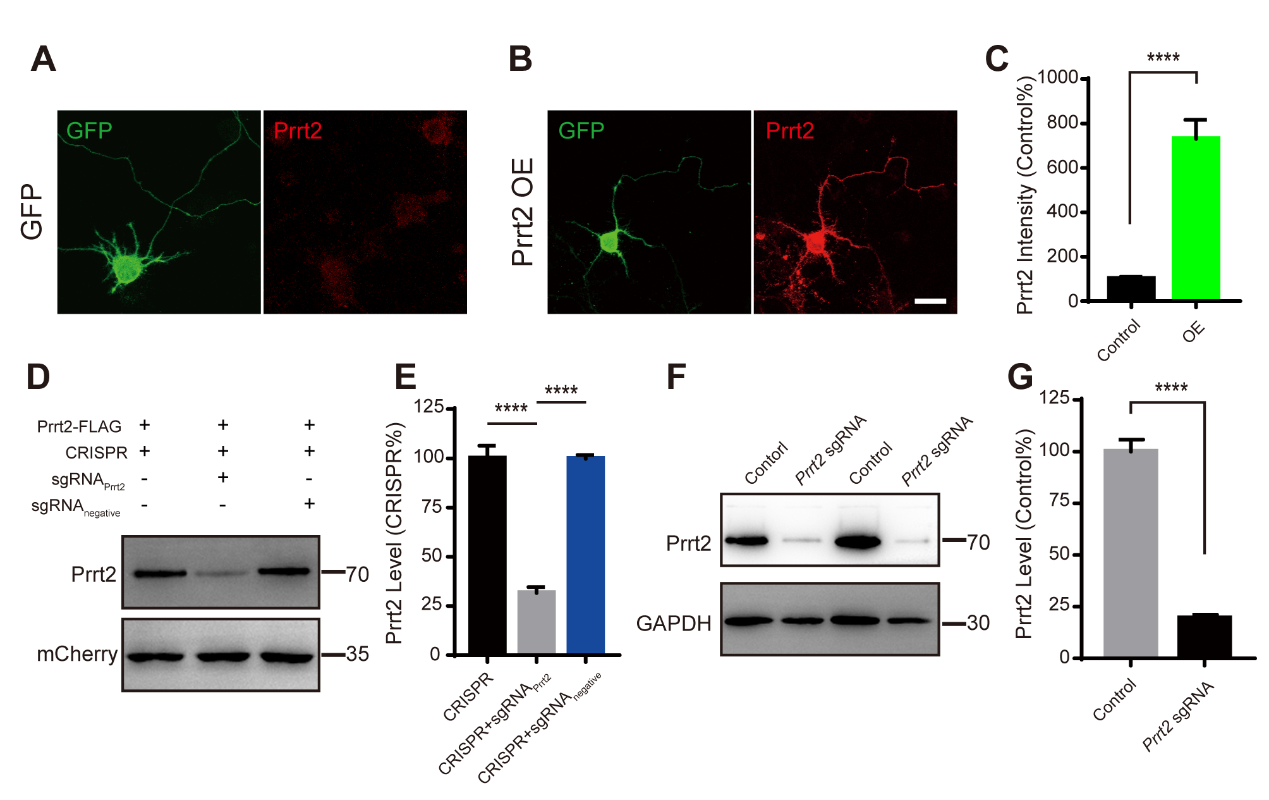


**Figure S2.** Prrt2 overexpression (OE) and knockout (KO) efficiency with the CRISPR-Cas9 system *in vitro* and *in vivo*. **(A, B)** Verification of OE efficiency using immunofluorescence with primary neuronal cultures transfected with green fluorescent protein (GFP) (as Control group) vectors or Prrt2 OE plasmids (as OE group) (Fig. 3A). **(C)** Immunofluorescence intensity analysis indicates high Prrt2 OE efficiency *in vivo* (****P<0.001 Control vs. OE). Scale bar: 20 μm. **(D)** Verification of KO efficiency using cell extracts from HEK 293T cells co-transfected with a variety of plasmids expressing spCas9, FLAG-tagged Prrt2, and Prrt2 or negative control single-guide (sg)RNA. **(E)** Western blotting results indicate the high Prrt2 KO efficiency of the CRISPR-Cas9 system *in vitro* (****P<0.001 CRISPR vs. CRISPR+sgRNA_Prrt2_; ****P<0.001 CRISPR+sgRNA_negative_ vs. CRISPR+sgRNA_Prrt2_; n=3). **(F)** Prrt2 protein levels in primary neuronal cultures infected with *Prrt2* sgRNA-expressing lentivirus and untransfected controls (as Control group). **(G)** Western blotting results indicate the high Prrt2 KO efficiency of the CRISPR-Cas9 system *in vivo* (****P<0.001 Control vs. *Prrt2* sgRNA; n=3).
